# Amebiasis in Mexico, 2014–2023

**DOI:** 10.3201/eid3102.241507

**Published:** 2025-02

**Authors:** Alberto Antonio-Campos, Keity J. Farfán-Pira, Alfonso D. Díaz-Fonseca, Carlos Ochoa-Velasco, Paola Hernández-Carranza, Diana M. Torres-Cifuentes

**Affiliations:** Instituto Politécnico Nacional, Mexico City, Mexico (A. Antonio-Campos); University of Arkansas, Fayetteville, Arkansas, USA (K.J. Farfán-Pira); Benemérita Universidad Autónoma de Puebla, Puebla, Mexico (A.D. Díaz-Fonseca, C. Ochoa-Velasco, P. Hernández-Carranza, D.M. Torres-Cifuentes)

**Keywords:** Amebiasis, public health, poverty, prevalence, Entamoeba histolytica, parasites, Mexico

## Abstract

Amebiasis remains a public health challenge in Mexico, especially in areas with poor sanitation. Despite declining prevalence (2014–2023), hotspots persist because of socioeconomic factors such as poverty. Addressing regional disparities through targeted interventions, improved infrastructure, and education is crucial to further reduce the disease burden and prevent future outbreaks.

Amebiasis, caused by *Entamoeba histolytica* protozoan parasites, leads to ≈100 million annual cases worldwide, particularly in developing regions (*1*). About 90% of infections are asymptomatic, creating reservoirs that perpetuate community transmission. The 10% of symptomatic infections cause dysentery, diarrhea, abdominal pain, and fever, often progressing to invasive intestinal amebiasis (IIA), which invades the intestinal mucosa and damages intestinal walls (*2*). Some cases advance to extraintestinal forms, such as amebic liver abscess (ALA) (*3*). We studied the epidemiology of amebiasis in Mexico, where the disease remains a public health challenge, emphasizing the need for monitoring programs in high-risk regions to promptly manage cases and complications.

## The Study

To analyze amebiasis trends in Mexico during 2014–2023, we examined IIA cases reported by healthcare providers to the Mexican Ministry of Health through the national epidemiologic surveillance system. We calculated prevalence per 100,000 inhabitants by using population data estimated from polynomial equations based on demographic data provided by the Instituto Nacional de Estadística y Geografía (Aguascalientes, Mexico). Reports were based on annual total confirmed cases recorded during the final week of each year (week 52 or 53); totals were summed for patients of both sexes. We evaluated the influence of socioeconomic factors and regional disparities on amebiasis by using 2015 IIA data, focusing on key population characteristics such as poverty and access to piped water and drainage (Appendix).

**Figure 1:**
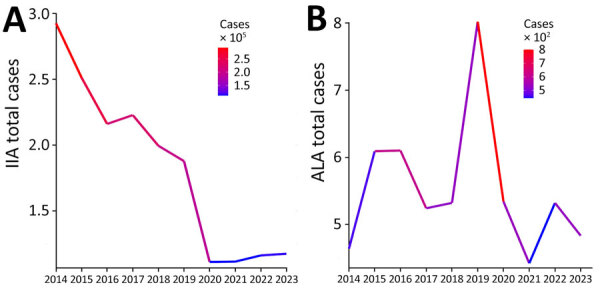
Annual prevalence (cases/100,000 inhabitants) of IIA (A) and ALA (B) in Mexico, by state, 2014–2023. ALA, amebic liver abscess; IIA, intestinal invasive amebiasis.

In 2014, the state of Hidalgo reported the highest number of IIA cases (2,535), 3.8 times that of Yucatan, the state reporting the second highest number of cases (Appendix Figure 1). Nationally, prevalence declined from 14.1% in 2014 to 5.86% in 2019 (Figure 1, panel A), potentially influenced by public health measures during the COVID-19 pandemic, as reported for other infectious diseases (*4*). Despite the decline, states such as Campeche, Chiapas, Guerrero, Hidalgo, Nayarit, Oaxaca, and Yucatan remain hot spots (Appendix Figure 1). During 2014–2023, a steady decrease in IIA cases indicated a reduction in illness (Appendix Table). Annual IIA case reports were as follows: 292,811 cases in 2014 (week 53), 251,416 in 2015 (week 52), 216,103 in 2016 (week 52), 222,813 in 2017 (week 52), 199,482 in 2018 (week 52), and 187,785 in 2019 (week 52). Declines in subsequent years were 111,054 cases in 2020, 111,284 in 2021, 116,012 in 2022, and 117,274 in 2023 (Figure 1, panel A).

ALA trends during 2014–2022 showed variability; prevalence peaked in Colima (7.79) and remained consistently high in Chihuahua and Sonora (Appendix Figure 2). National ALA cases increased until 2019, followed by a decline through 2023. Reported cases were as follows: 490 cases in 2014 (week 53), 609 in 2015 (week 52), 610 in 2016 (week 52), 524 in 2017 (week 52), 532 in 2018 (week 52), and a peak of 802 cases in 2019 (week 52). Subsequently, cases decreased to 540 in 2020 (week 53), 435 in 2021 (week 52), 528 in 2022, and 481 in 2023 (week 52) (Figure 1, panel B; Appendix Table). High-prevalence states such as Colima, Nayarit, Sinaloa, Sonora, and Oaxaca face persistent challenges, underscoring the need for improved hygiene and food safety.

Amebiasis exhibits demographic patterns in Mexico. Women are more frequently affected by IIA (Appendix Figure 3, panel A), whereas ALA is 10 times more prevalent among men (Appendix Figure 3, panel B).

**Figure 2:**
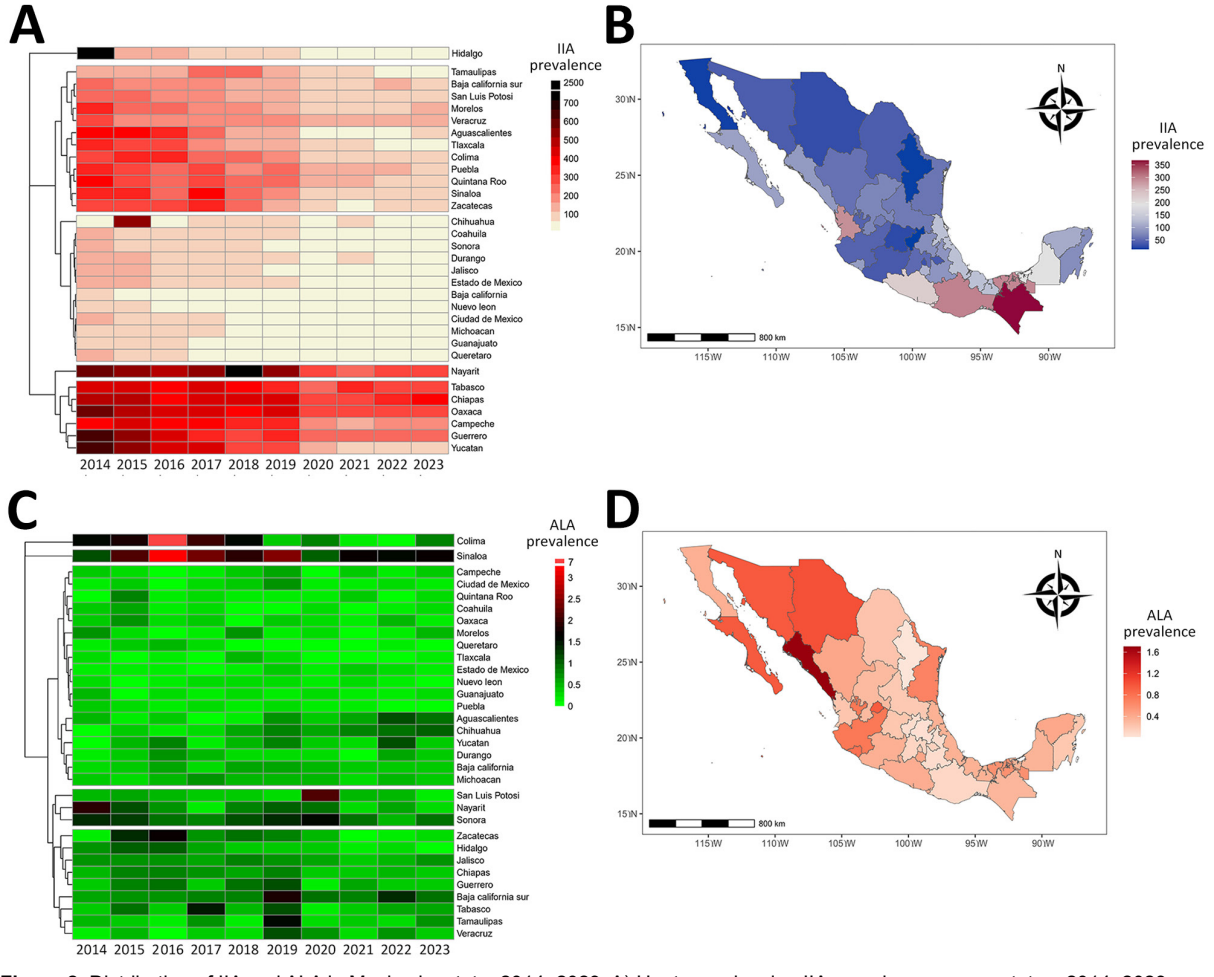
Distribution of IIA and ALA in Mexico by state, 2014–2023. A) Heatmap showing IIA prevalence across states, 2014–2023. B) Geographic distribution of IIA prevalence by state in 2023. C) Heatmap showing ALA prevalence across states, 2014–2023. D) Geographic distribution of ALA prevalence by state in 2023. ALA, amebic liver abscess; IIA, intestinal invasive amebiasis.

During 2014–2023, southern states such as Nayarit, Tabasco, Chiapas, Oaxaca, Campeche, Guerrero, and Yucatan consistently reported high IIA prevalence (Figure 2, panels A, B). Conversely, ALA cases predominated in northern states, including Sinaloa, Sonora, Chihuahua, and Baja California, and in the central regions such as Nayarit and Aguascalientes; prevalence ranged from 0.96 to 1.70 (Figure 2, panels C, D). Of note, Nayarit and Sinaloa reported high prevalence for both IIA and ALA, highlighting substantial public health burdens.

Amebiasis disproportionately affects low-income areas (*5*). Analysis of 2015 IIA data revealed high prevalence, especially in rural regions with poor sanitation (e.g., Nayarit, Chihuahua, Yucatan, Guerrero, and Chiapas) (Appendix Figure 4, panel A). Piped water and drainage were low in states such as Chiapas, Guerrero, Oaxaca, San Luis Potosi, and Veracruz (Appendix Figure 4, panel B), and poverty exceeded 60% in regions such as Ciudad de Mexico, Guerrero, Oaxaca, and Puebla (Appendix Figure 4, panel C). The association between poverty and IIA prevalence was statistically significant (r = 0.46, p<0.01) (Appendix Figure 4, panel D).

## Conclusions

Amebiasis remains a substantial health issue in Mexico, primarily because of its ease of transmission and associated illness and death rates. The US National Institute of Allergy and Infectious Diseases (Bethesda, MD, USA) classifies *E. histolytica* as a category B agent, underscoring the urgent need for enhanced diagnostic tools and surveillance systems (*6*,*7*). The consistently high prevalence of IIA in southern Mexico aligns with previous reports of elevated *E. histolytica* antibody levels compared with northern Mexico (*8*).

Of note, women are more frequently affected by IIA and men are more frequently affected by ALA. That difference may be attributed to factors such as hormonal effects, including the protective effect of iron-deficiency anemia or other hormonal factors in women of childbearing age (*9*) and potential increased susceptibility in men because of alcohol-associated liver damage (*10*).

Addressing the variable prevalence of ALA across Mexico is crucial. The complication is endemic to tropical regions but is increasingly reported globally, probably because of factors such as international travel and transmission through oral–anal contact (*1*). Although amebiasis primarily affects children in developing countries, the absence of detailed age-specific data from the Mexican Ministry of Health limits development of targeted interventions.

Our findings indicate an inverse relationship between IIA and ALA cases in Mexico, particularly in the southern regions. Previous studies suggest that 2%–5% of IIA cases progress to ALA and that genetic factors, such as specific alleles in the major histocompatibility complex (HLA-DRB1 and HLA-DQB1), play a critical role in resistance or susceptibility to ALA (*11*). That relationship is further complicated by regional differences in healthcare access, diagnostic practices, and reporting standards. ALA cases may be more readily identified in areas with advanced medical facilities, and IIA may be underreported in regions with limited infrastructure.

Although we found no direct link between sanitation and IIA prevalence, our analysis revealed a moderate correlation with socioeconomic conditions, which underscores the role of factors such as poverty, access to clean water, and sanitation infrastructure in the burden of intestinal parasitic infections (*12*). Similar trends have been observed in other countries, including Malaysia (*13*), Iraq (*14*), and Ethiopia (*15*). Sociodemographic factors such as low income, inadequate sewage systems, and domestic animal waste contribute substantially to the prevalence of enteric infections. Enhanced hygiene practices and educational initiatives can play a crucial role in reducing prevalence.

In conclusion, amebiasis continues to pose a substantial health challenge in Mexico. Strengthening sanitation, health education, and surveillance efforts is essential. Ongoing research into virulence mechanisms and socioeconomic effects of the disease will support development of better treatment and prevention strategies and ultimately address the complex public health issues more effectively.

Appendix Additional information for study of amebiasis in Mexico, 2014–2023.
