# Supplementary material for: Amebiasis in Mexico, 2014–2023
Source: Emerg Infect Dis. 2025 Feb;31(2):359–62. doi: 10.3201/eid3102.241507 (PMC11845125; doi:10.3201/eid3102.241507)
Supplement: Appendix — Additional information for study of amebiasis in Mexico, 2014–2023. [file 24-1507-Techapp-s1.pdf]

Article DOI: https://doi.org/10.3201/eid3102.241507

EID cannot ensure accessibility for supplementary materials supplied by authors. Readers who have difficulty accessing supplementary content should contact the authors for assistance.

Amebiasis in Mexico, 2014–2023

Appendix

Methods appendix and sources of data

Data collection

Information was manually extracted from the epidemiologic bulletins published by the Mexican Ministry of Health, accessible through their open access portal: https://www.gob.mx/salud/acciones-y-programas/historico-boletin-epidemiologico. Data from the final reported week of each year were collected for invasive intestinal amoebiasis (IIA) and amebic liver abscess (ALA). Total case counts for patients of both sexes were summed for each state.

Population data

Population figures were obtained from the Instituto Nacional de Estadística y Geografía (Aguqscalientes, Mexico) via https://www.inegi.org.mx, referencing census data from 2000, 2005, 2010, 2015 and 2020. For the years 2014, 2016, 2017, 2018, 2019, 2021, 2022 and 2023, population estimates were generated using fourth-degree polynomial equations, derived with the aid of WolframAlpha (Polynomial Regression Widget): https://www.wolframalpha.com. Prevalence rates were calculated by dividing total case counts by the estimated population for each year and standardized per 100,000 individuals.

Geographic data

The geographic coordinates for Mexican states were sourced from the Comisión Nacional para el Conocimiento y Uso de la Biodiversidad (Mexico City, Mexico) via http://www.conabio.gob.mx, ensuring accurate regional mapping.

Prevalence and percentage change analysis

Percentage changes in prevalence of Appendix Table were calculated using the following equation: $\frac{(2023\ prevalence - 2014\ prevalence)}{2014\ prevalence} x\ 100$. This analysis compares IIA and ALA prevalence across states from 2014 to 2023, highlighting regional disease trends.

Socioeconomic and environmental data

Data information on the percentage of inhabited dwellings with access to piped water and drainage systems (2015) was obtained from the Instituto Nacional de Estadística y Geografía (Aguascalientes, Mexico) via https://www.inegi.org.mx/temas/vivienda.

Poverty rates were sourced from the Instituto Nacional de Estadística y Geografía interactive tables: https://www.inegi.org.mx/temas/ingresoshog/#informacion_general. Annual temperature data for 2022 were accessed through the National Meteorological System https://smn.conagua.gob.mx, and climatological reports: https://smn.conagua.gob.mx/tools/DATA/Climatología/Pronóstico%20climático/Temperatura%20y%20Lluvia/TMED/2022.pdf

Statistical analysis

Normality testing: The Shapiro-Wilk test was employed to assess data distribution.

Normalization: Data were normalized using the Z-Score method.

Correlation analysis: Spearman's rank correlation was performed to examine relationships between IIA prevalence, poverty levels, and access to infrastructure.

Visualization tools

Data visualization was conducted in R version 4.1.2 (https://www.r-project.org/) and R-Studio version 4.1.2 (https://www.rstudio.com/). The packages used include ggplot2, pheatmap, and pairs.panel.

Appendix Table. Annual prevalence/100,000 inhabitants of IIA and ALA in Mexico by state, 2014 and 2023, along with the percentage change, highlighting regional dynamics and disease trends

	IIA			ALA		
State	2014	2023	% change	2014	2023	% change
Aguascalientes	409	63	−85	0.47	0.96	104
Baja California	77	15	−81	0.30	0.37	23
Baja California Sur	233	99	−58	0.57	0.96	68
Campeche	408	189	−54	0.34	0.34	0
Coahuila	126	40	−69	0.38	0.24	−37
Colima	274	96	−65	1.57	0.81	−49
Chiapas	489	368	−25	0.47	0.33	−30
Chihuahua	28	26	−8	0.00	1.02	-
Ciudad de Mexico	116	31	−74	0.11	0.19	72
Durango	161	56	−66	0.17	0.43	152
Guanajuato	103	23	−78	0.45	0.22	−52
Guerrero	635	225	−65	0.34	0.39	14
Hidalgo	2535	81	−97	0.74	0.06	−92
Jalisco	142	45	−69	0.75	0.76	1
Estado de Mexico	157	50	−69	0.16	0.13	−19
Michoacan	109	37	−67	0.35	0.37	5
Morelos	337	118	−65	0.74	0.45	−40
Nayarit	614	287	−54	1.88	0.23	−88
Nuevo Leon	90	14	−85	0.24	0.02	−92
Oaxaca	604	299	−51	0.33	0.11	−67
Puebla	323	86	−74	0.38	0.07	−82
Queretaro	117	13	−89	0.20	0.07	−65
Quintana Roo	366	80	−79	0.07	0.22	214
San Luis Potosi	237	63	−74	0.48	0.21	−57
Sinaloa	326	93	−72	1.19	1.70	42
Sonora	128	40	−69	1.38	0.96	−31
Tabasco	446	295	−34	0.38	0.59	55
Tamaulipas	120	59	−51	0.44	0.68	54
Tlaxcala	327	36	−89	0.24	0.15	−38
Veracruz	272	131	−52	0.21	0.37	76
Yucatan	661	109	−84	0.10	0.43	330
Zacatecas	278	73	−74	0.13	0.30	130

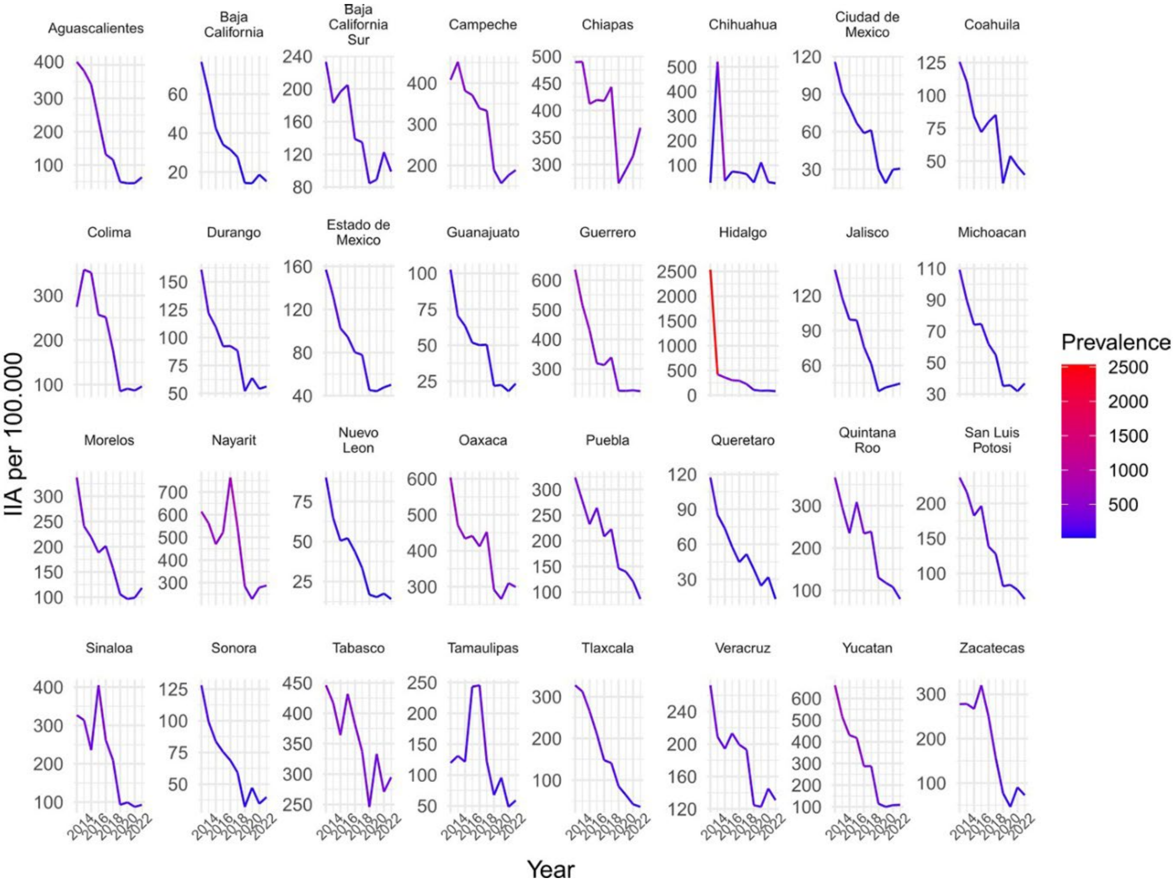

Appendix Figure 1. Total IIA cases in Mexico, 2014-2023. Annual prevalence/100,000 inhabitants. IIA, intestinal invasive amebiasis.

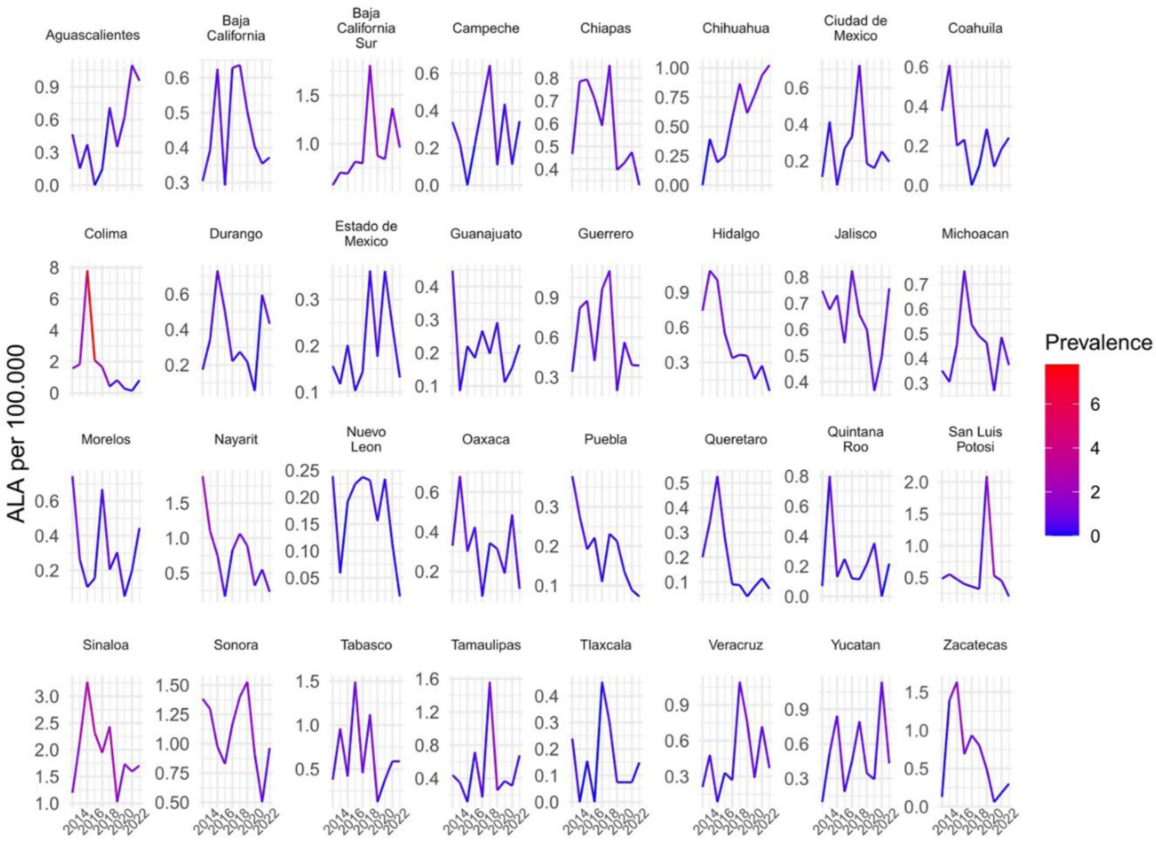

Appendix Figure 2. Total ALA cases in Mexico, 2014-2023. Annual prevalence/100,000 inhabitants. ALA, amebic liver abscess.

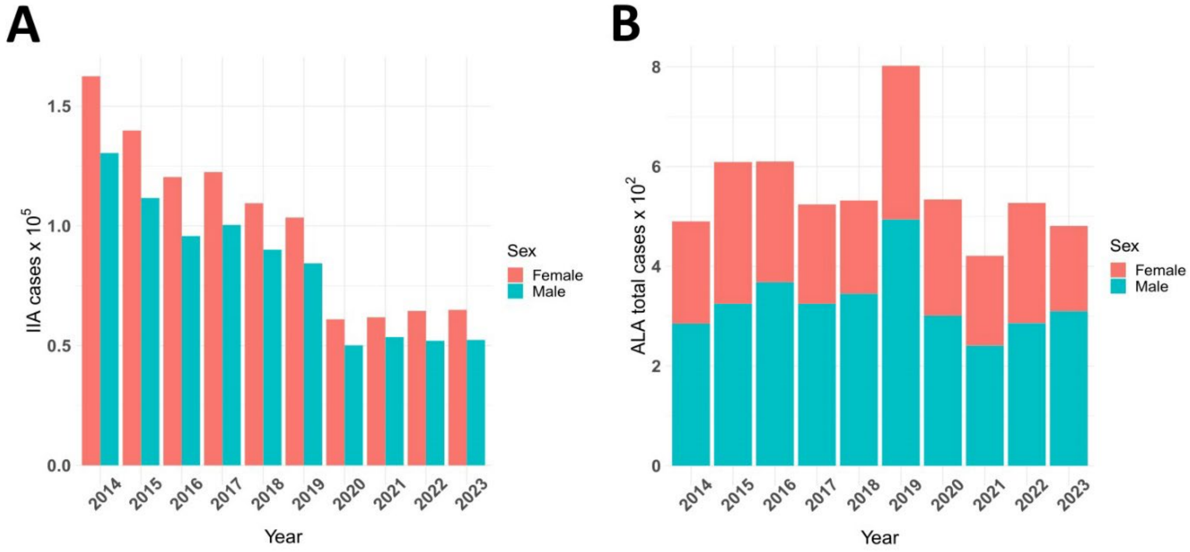

Appendix Figure 3. Sex-specific trends of IIA and ALA, 2014-2023. A) Annual IIA cases/100,000 inhabitants, differentiated by sex. B) Annual ALA cases/1,000 inhabitants, differentiated by sex. IIA, intestinal invasive amebiasis; ALA, amebic liver abscess.

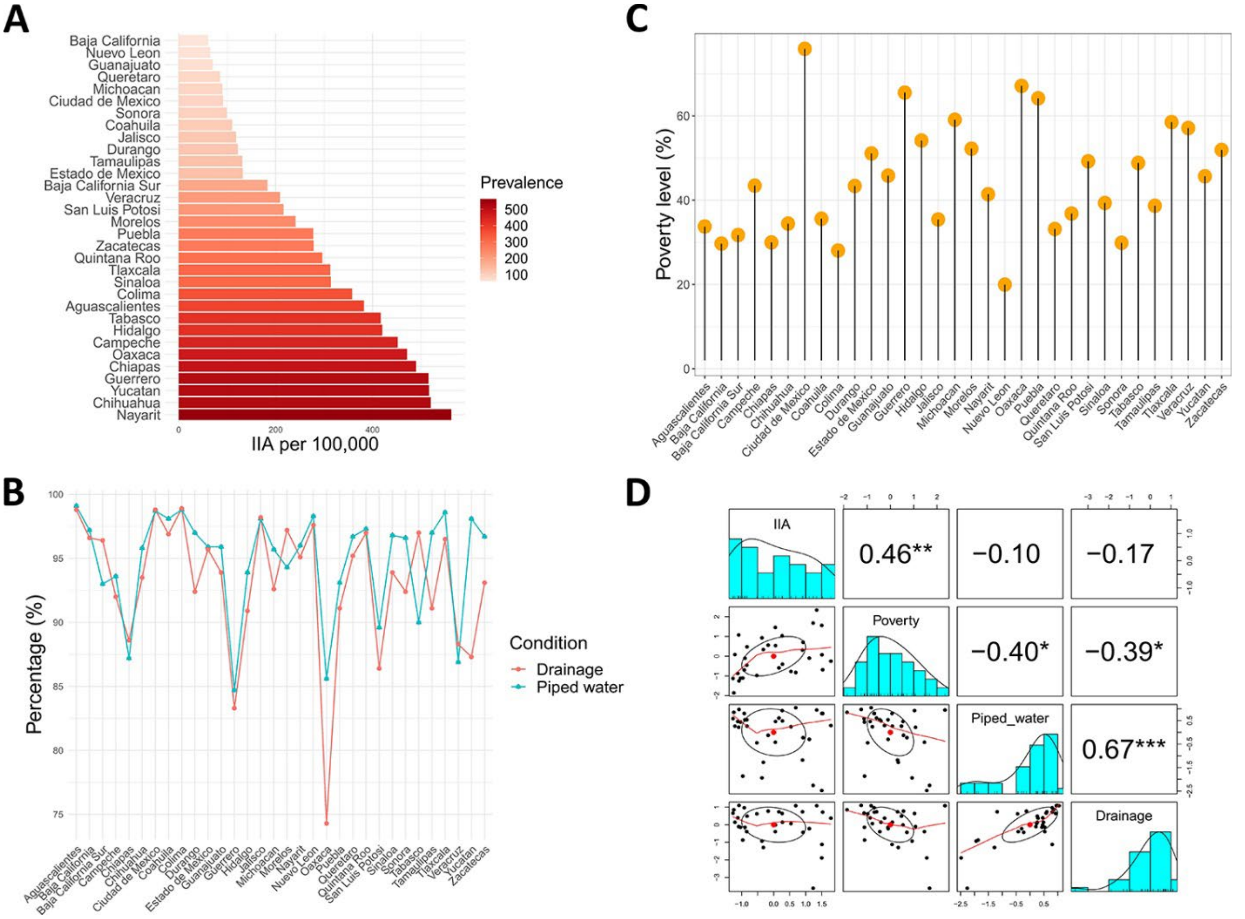

Appendix Figure 4. Epidemiologic analysis of IIA and its relationship with socioeconomic factors in Mexico, 2015. A) IIA prevalence by state in 2015. B) Percentage of inhabited private dwellings with access to piped water and drainage systems in 2015. C) Percentage of the population living in poverty in 2015. D). Correlation analysis between IIA prevalence, poverty levels, and access to piped water and drainage systems in 2015. The panel presents a pairwise correlation matrix, depicting the relationships between IIA prevalence, poverty, access to piped water, and drainage systems. Scatter plots show data from a specific Mexican state. Red line indicates the trend, and ellipse, data dispersion. Numerical values represent the correlation coefficient, where positive values suggest a direct relationship, while negative values imply an inverse relationship. Statistical significance is indicated as *p < 0.05; **p < 0.01; ***p < 0.001. IIA, intestinal invasive amebiasis.
